# Effects of active forest management on host-seeking tick density and infection prevalence: a systematic review and meta-analysis

**DOI:** 10.1098/rspb.2025.0851

**Published:** 2025-10-29

**Authors:** Stephanie N. Hurd, Elissa S. Ballman, Jessica E. Leahy, Megan L. Schierer, Allison M. Gardner

**Affiliations:** ^1^ Department of Biology, University of Massachusetts, Boston, MA 02125, USA; ^2^ School of Biology and Ecology, University of Maine, Orono, ME 04469, USA; ^3^ School of Forest Resources, University of Maine, Orono, ME 04469, USA

**Keywords:** tick-borne pathogen, prescribed burn, invasive vegetation, invasive plant, exotic shrub, timber harvesting, systematic literature review, meta-analysis

## Abstract

Hard-bodied ticks (Acari: Ixodidae) pose a major public health threat, transmitting multiple pathogens among humans and wildlife worldwide. Research has investigated how anthropogenic land use change impacts tick density and infection prevalence in temperate forests, including the effects of active forest management practices like prescribed burning, invasive vegetation removal and timber harvesting. However, studies’ results are inconsistent and seemingly context-dependent, making it difficult for land managers, landowners and policy makers to identify whether management addresses the public health concern. We performed a systematic review and meta-analysis and documented a net decrease in tick density correlating with prescribed burns and invasive vegetation removal, but no effect on tick infection prevalence. Our review of a substantially smaller number of timber harvesting-focused studies showed the same pattern. Through a systematic literature review, we explored potential causal pathways between these management practices and lower tick density, including microclimate- and host-driven mechanisms. We recommend that future research explore mechanisms for tick infection prevalence and, for prescribed burn studies, employ standardized measurements of burn intensity and consider long-term effects post-burn.

## 1. Introduction

Hard-bodied ticks (Acari: Ixodidae) transmit numerous pathogens among humans and wildlife and facilitate tick-borne disease expansion [1–3]. To combat this threat to public health, identifying strategies to control tick populations has emerged as a critical research focus. In particular, most management efforts aim to reduce the density of infected ticks—defined as the product of density of ticks and tick infection prevalence—which is the widely accepted measure for approximating the likelihood of encountering an infected tick in the environment (‘entomological risk’) [4]. Prior research has found vegetation management, including active forest management conducted by land managers and landowners, a socially acceptable [5] and potentially environmentally sustainable approach to reduce tick density and infection prevalence. In temperate forests, prescribed burning, removal of invasive vegetation and timber harvesting could help mitigate threats to public health posed by multiple pathogens transmitted by vector species including, in North America, the blacklegged tick (*Ixodes scapularis*), western blacklegged tick (*I. pacificus*), the American dog tick (*Dermacentor variabilis*) and the lone star tick (*Amblyomma americanum*). This systematic review and meta-analysis synthesizes the findings of prior field studies spanning a range of tick-borne disease systems to consider the net effects of these three management practices on tick density and infection prevalence.

While numerous studies have examined consequences of prescribed burns for tick density and infection prevalence, the direction and magnitude of treatment effects are inconsistent. The few studies measuring how timber harvesting impacts ticks reported reduced *Ixodes* spp. densities in harvested forests [6–8], but this relationship remains understudied. Several studies investigating tick densities on burned plots compared with unburned control plots found that prescribed burns reduce densities of *I. scapularis* [9–11] and *A. americanum* [12]. However, Gilliam *et al*. [13] found no effect of burning on *A. americanum* abundance. Additionally, there is evidence of an effect of time since burning; prescribed fires may initially reduce *A. americanum* densities followed by a population rebound 2 to 5 years later that exceeds unburned areas [14,15]. Results from invasive vegetation removal studies are also inconsistent. Some studies found plots with an absence or removal of invasive vegetation like Japanese barberry (*Berberis thunbergii*), Eurasian honeysuckle (*Lonicera* spp.) or common buckthorn (*Rhamnus cathartica*) had lower *I. scapularis* densities compared with invaded plots [16–19]. A study conducted in Delaware found that, within the same forest fragment invaded with multiflora rose (*Rosa multiflora*), *I. scapularis* densities were higher in subplots containing *R. multiflora* than subplots where this invasive plant was absent. Yet in comparing invaded versus uninvaded forest fragments 6 to 16 ha in area, nymph densities were higher in forest fragments without *R. multiflora* [20]. Reported effects of invasive vegetation removal on tick infection prevalence are inconsistent and potentially nuanced depending on tick and invasive vegetation species [21–23].

Quantifying the impacts of prescribed burns, invasive vegetation removal and timber harvesting on tick density and infection prevalence is important to land managers, landowners and policy makers to identify under what conditions these management practices effectively mitigate tick-borne disease exposure risk, thereby benefitting public health. The goal of our meta-analysis is to standardize the reported effects from individual studies to explore patterns that extend beyond the scope of individual field studies, transcending study design considerations such as sample size, tick genus examined and study location. Our meta-analysis contributes to a growing body of research that investigates the consequences of anthropogenic land use change for tick populations and identifies potential mechanisms and modifying factors explaining relationships between forest structure and vectors. The active forest management practices that we consider are often used to meet property owners’ goals that are unrelated to tick-borne disease mitigation, including burning for wildfire hazard abatement [24,25], removing invasive plants to promote endangered or endemic species’ recovery and native biodiversity [26,27], and harvesting timber to realize income, promote regeneration, minimize insect and disease damage, or improve recreation [28,29]. Understanding the consequences of these management practices for tick populations enables us to identify situations in which best practices for public health are complementary to landowners’ other goals for their forested land.

In this study, we compare the effects of prescribed burns, invasive vegetation removal and timber harvesting in temperate forests on host-seeking hard-bodied tick density and infection prevalence. We then perform a systematic literature review to explore potential biotic and abiotic pathways underlying these effects. The objectives of this study are (i) to estimate the overall effects of prescribed burns, invasive vegetation removal, and timber harvesting on tick density and infection prevalence, (ii) to identify and characterize factors that contribute to between-study heterogeneity and overall effect estimates, and (iii) to explore potential mechanisms explaining any patterns observed in objectives (i) and (ii).

## 2. Material and methods

### (a) Literature search

We performed a systematic literature review and meta-analysis following the Preferred Reporting Items for Systematic Reviews and Meta-Analyses (PRISMA) guidelines [30] (electronic supplementary material, File 1, table S1). We identified relevant peer-reviewed articles published through 12 September 2023, conducting searches on the Web of Science and PubMed databases on 12 and 13 September 2023, respectively. We used the keywords: ‘tick* AND burn’; ‘tick* AND invasive* AND plant*’; ‘tick* AND invasive* AND vegetation*’; ‘tick* AND invasive* AND shrub*’; ‘tick* AND shrub*’; ‘tick* AND timber* AND harvest*’; and ‘tick* AND logging*’. Our inclusion criteria included peer reviewed empirical studies published in English and conducted within temperate forests in North America and Europe, with a focus on the impact of (i) prescribed (i.e. controlled or managed) fires, (ii) invasive vegetation removal or comparisons of invasive vegetation presence versus absence, or (iii) timber harvesting on hard-bodied tick species (Acari: Ixodidae). We excluded studies on soft-bodied tick species (Acari: Argasidae), theoretical models, laboratory studies, review studies, studies conducted in different habitats like savannas, grasslands or prairies, studies on wildfires, studies examining the effects of combined management practices, and studies published more than 50 years ago. We restricted the search to host-seeking (‘questing’) ticks and excluded studies of on-host ticks as per CDC recommendations [31]. These measures of tick abundance provide different information about the ecology of the system so it is not standard to directly compare them (e.g. on-host tick collection does not inform on the density of nymphs in an area, while questing tick collection can) [31].

Electronic supplementary material, figure 1 describes the steps of the systematic article selection process. Our database searches returned 492 articles from Web of Science and 259 from PubMed, for a total of 751 articles (including overlap between both databases) (see electronic supplementary material, File 2, table S2). Two readers, working independently, assessed the titles and abstracts without any additional article information, including blinding the reviewers to the identity of the study authors when possible. From the Web of Science search, one reader (S.N.H.) reviewed all titles and abstracts and the second (M.L.S.) reviewed 325 (43.28%). One reader (S.N.H.) reviewed all titles and abstracts from the PubMed search. Assessing reader agreement ensures that the eligibility screening process is reproducible and demonstrates whether the search criteria need refinement; a Cohen’s kappa analysis that compares actual reader agreement with agreement expected by chance alone [32,33] found kappa = 0.75, indicating substantial reader agreement [34,35]. If at least one reader identified an abstract as relevant it was included for full-text review. From the original 751 articles returned from both database searches, 79 were retained for full-text review after the removal of articles owimg to exclusion criteria, repeats from different keyword searches or duplicates from each search database (electronic supplementary material, File 3, table S3). Two authors (S.N.H. and E.S.B.) conducted the full-text review, working independently, to sort the articles into irrelevant versus relevant papers to include. One author (S.N.H.) read all full-text articles and the second (E.S.B.) read 40 (50.63%). We again performed a kappa analysis and determined that the full-text co-review process passed the assessment (kappa = 0.75). We returned to the five articles where there was co-reviewer decision discrepancy and reached a consensus after revisiting exclusion criteria. Post full-text review, we originally selected 25 articles to include in the review. Upon further examination, we excluded four additional articles—three because we determined that they were not conducted within temperate forests, and one because the authors cautioned against comparing burned versus unburned conditions owing to inconsistent habitat types in the study design. Ultimately, we included 21 articles total in the systematic review and meta-analysis (electronic supplementary material File 4, table S4). Of this list, only two [6,7] investigated the effects of timber harvesting. While we summarize findings from these two papers in §3, we excluded these timber harvesting studies from the formal meta-analysis because this sample size is too small to conduct robust statistical analyses.

### (b) Calculating effect sizes

To compare the effects of each management practice on tick densities and infection prevalence, we extracted effect sizes and estimates of precision (i.e. variance) from 21 studies (electronic supplementary material, File 5). We extracted paired means to estimate effect size, or the standardized difference among means, using Hedge’s *g* [36] as described in Ellis [37] and Koricheva *et al*. [38]. The summary statistics needed to calculate *g* are sample mean (Ȳ), sample standard deviation (SD) and sample size (*n*) for each group under comparison. If the SD was not provided, we back-calculated it based on the standard error (SE) and sample size, when available.

We extracted paired means to compare control conditions versus treatment conditions. For the prescribed burn phase of the analysis, the ‘control’ groups were defined as areas where prescribed burns had not been conducted within the past decade; the ‘treatment’ groups were defined as areas where prescribed burns have been applied in the past decade. For the invasive vegetation phase of the analysis, the ‘control’ groups were defined as areas with intact invasive vegetation, while the ‘treatment’ groups were defined as areas where invasive vegetation was purposefully removed or naturally absent. We extracted paired control versus treatment means of questing tick density and/or infection prevalence from each study. The effect size for each study was determined by subtracting the treatment tick density or infection prevalence mean from the control tick density or infection prevalence mean. Therefore, positive effect sizes indicate that the management practice was associated with reduced tick density or infection prevalence.

If paired means were provided for multiple years following management, we included each pair in the analysis and recorded the time since management as a moderator. Other moderators included geography (i.e. the geographical location where the study occurred), tick species and life stage, area of the experimental units on which data collection occurred, forest stand type (i.e. oak, pine or mixed), burn intensity measured by flame height and/or burn frequency, invasive vegetation species present and management tool used (e.g. mechanical removal versus targeted flame removal of invasive plants). Some studies made observations across multiple spatial scales (e.g. [20,23]). In these instances, we only compared means that were calculated within the same spatial area and included the spatial scale as a moderator. As there was substantial variation in how authors described some of these moderators, we coded each study’s description to a defined group or along a defined range (electronic supplementary material, File 6, table S6).

When an article failed to report the mean, SD and sample size, efforts were made to retain the study to avoid compounding publication bias and increasing the possibility of making a review level type II error [38,39]. When necessary, we performed our own calculations from the raw data provided, visually extracted raw data from graphs, or performed algebraic recalculations on summary statistics like *t*-scores or *F*-scores [38]. In rare instances (*n* = 2) we were unable to extract data when neither relevant statistics nor raw data were reported. We included these articles only in the literature review.

### (c) Statistical analysis

To estimate the effect of prescribed burns and invasive vegetation removal on tick density and infection prevalence, we analysed the standardized mean difference between control and treatment conditions across all studies (hereafter referred to as the ‘overall effect’). We assumed there to be additional between-study heterogeneity outside of sampling error owing to different experimental conditions (e.g. geographical locations, tick species and life stages). Random effects models were used to estimate the amount of total heterogeneity ($\tau^{2}$) and its associated variance (SE); the Q-statistic is reported as a measure of the statistical significance of these moderating factors. We constructed four separate random effects models using the rma function from the metafor package [40] in R v. 4.3.1 [41] to test the effect of prescribed burns and invasive vegetation removal on tick density and infection prevalence, and to test for between-study heterogeneity. Each model included the study-specific effect estimate of the standardized mean difference of control versus treatment groups, Hedge’s *g*, and the associated measure of uncertainty that reflects within-study variability, Hedge’s variance, var(*g*). Data were normally distributed except for two models: prescribed burns and invasive vegetation removal versus tick density. For the burn versus tick density model, we assumed the effect estimate to be normally distributed, consistent with established findings that the Central Limit Theorem supports normal approximation when sample sizes are sufficiently large (*n* = 74 paired means) [42–44]. In the invasive vegetation removal versus tick density model the effect estimate was slightly skewed owing to the presence of outliers (upper outlier *g* = 4.89 and lower outlier *g* = −3.41) and a smaller sample size (*n* = 36 paired means). Removal of these outliers passed the Shapiro–Wilk’s test of normality (*W* = 0.95; *p* = 0.13), although the inclusion of outliers did not affect the conclusions of statistical tests. Therefore, we chose to report the results of the model with the outliers included. We fit the models using the restricted maximum likelihood (REML) method, which is less sensitive to deviations from normality [45,46].

To identify factors that contribute to overall effect estimates and between-study heterogeneity, we constructed separate mixed effects meta-regression models using the rma.mv function from the metafor package in R [40,41], with study characteristics as fixed or random effects. Our random effects models for prescribed burns and invasive vegetation removal versus tick infection prevalence did not provide evidence of substantial between-study heterogeneity. Therefore, we only constructed mixed effects models for the effects of forest management on tick density. To assess the net treatment effect across studies and the different geographical locations in which these studies were conducted, both models included study accession number (ACC #) and geography as random effects. The burn model also included forest stand type, spatial scale, time since management, and tick genus and life stage as fixed effects. The invasive vegetation removal model included the same moderators, as well as the management tool used and invasive plant genus as fixed effects, but we removed the time since management moderator because less than half of the observations (16 out of *n* = 36 paired means, 44%) contained this information. We also removed the spatial scale moderator as there was a large disparity in sample size between the two levels of this moderator: 35 paired means at the spatial scale of metres versus just one pair of means at 5−30 ha. Thus, with only a single datapoint for one of the levels, we could not robustly model the effects of this moderator. We included all moderators in these models *a priori* to control for potential confounding factors and heterogeneity among studies, rather than selecting or dropping certain moderators based on statistical significance. These analyses account for study-level clustering, treating multiple paired means from the same study as non-independent [40].

## 3. Results

### (a) Meta-analysis

#### (i) Prescribed burns

Eight studies on the effect of prescribed burns on tick density yielded 74 paired means. Only three studies also included assessments of tick infection prevalence, but one did not provide enough statistics and/or raw data to calculate effect sizes, thus resulting in a sample size of six paired means across two studies ([47]: 5 pairs; [48] : 1 pair). The application of prescribed burns correlated with reduced tick density (estimated overall effect = 0.31, SE = 0.08, *p* < 0.01), but not infection prevalence (estimated overall effect = 0.13, SE = 0.26, *p* = 0.61) (table 1, electronic supplementary materials, figures 2 and 3). While significant, the effect size was moderate, indicating that tick density is lower in areas of prescribed burns, but not necessarily drastically lower. For example, if an unburned area has a mean tick density of 25 ticks per 100 m^2^ (SD = 10), tick density in a burned area should be lower by 3.1 ticks for a mean of 21.9 ticks per 100 m^2^. This effect may become more ecologically meaningful when management is applied over larger areas; using this same example, mean tick density in a hectare of burned area could be lower by 310 ticks compared with an unburned hectare.

**Table 1.** Results of the four random-effects models comparing standardized tick density and infection prevalence means of unmanaged controls with managed treatments via prescribed burns and invasive vegetation removal. Asterisk denotes significance at *α* = 0.05.

	Burn vs. Tick Density	Invasive vs. Tick Density	Burn vs. Tick Infection Prevalence	Invasive vs. Tick Infection Prevalence
Overall Mean Difference Across Studies				
Estimate	0.31	1.01	0.13	0.28
Variance (SE)	0.08	0.18	0.26	0.18
*p*‐value	0.0001*	<0.0001*	0.61	0.11
95% CI	0.15, 0.47	0.67, 1.36	–0.37, 0.63	–0.07, 0.64
Heterogeneity				
Estimate (tau^2^)	0.40	0.78	0	0
Variance (SE)	0.08	0.26	0.25	0.17
Test: Q-statistic, df	418.88, 73	265.09, 35	6.75, 5	11.81, 13
Test: *p*‐value	<0.0001*	<0.0001*	0.24	0.54

Estimates of between-study heterogeneity were substantial for the tick density model, but not for the tick infection prevalence model (table 1). Together, the moderators did significantly influence variation in effect sizes (*Q*_*M*_ = 30.89, *df* = 11, *p* = 0.001). The only significant single moderator was time since management, though the effect size was small (table 2). This model explained 84% of between-study variance compared with the intercept-only model. However, the test for residual heterogeneity was significant (*Q*_*E*_ = 308.28, *df* = 62, *p* < 0.0001), indicating that other factors or levels of moderators not considered in the model likely influenced the impact of prescribed burns on tick density.

**Table 2.** Results of the two mixed-effects models assessing whether certain moderators account for between-study heterogeneity when assessing the effect of prescribed burns and invasive vegetation removal on tick density. The moderators included in the models were the random effects of study accession number and geography, and the fixed effects of time since management, tick genus and life stage, forest stand type, experimental unit size, tool used and invasive genus. Reference categories were *Amblyomma* for tick genus, adult for tick life stage, mixed for forest stand type, metres for spatial scale, targeted flame for tool used in invasive vegetation removal and *Berberis* for invasive vegetation genus. The estimate values are estimates of the overall mean effect across all levels of each moderator. Asterisk denotes significance at *α* = 0.05.

	Burn Management vs. Tick Density Model Results					Invasive Management vs. Tick Density Model Results					
Moderator	Estimate	Variance (SE)	*z*-value	*p*‐value	95% CI	Estimate	Variance (SE)		*z*-value	*p*‐value	95% CI
Intercept	–2.19	1.32	–1.67	0.10	–4.77, 0.40	2.22	2.13	1.05		0.30	–1.94, 6.39
Time Since Management	0.04	0.01	2.74	0.01*	0.01, 0.06	NA	NA	NA		NA	NA
Tick genus (*Dermacentor*)	0.65	1.05	0.62	0.54	–1.41, 2.72	NA	NA	NA		NA	NA
Tick genus (*Ixodes*)	1.04	0.55	1.89	0.06	–0.04, 2.12	0.15	2.14	0.07		0.94	–4.04, 4.35
Tick life stage (Larvae)	–0.07	0.04	–1.82	0.07	–0.15, 0.01	NA	NA	NA		NA	NA
Tick life stage (Nymph)	–0.04	0.04	–0.99	0.32	–0.10, 0.03	–0.35	0.12	–3.04		0.002*	–0.58, –0.13
Tick life stage (Pooled)	1.40	1.20	1.16	0.25	–0.96, 3.75	–3.19	1.55	–2.07		0.04*	–6.22, –0.16
Forest stand type (Oak)	2.06	1.46	1.41	0.16	–0.80, 4.92	–0.67	1.66	–0.40		0.69	–3.92, 2.59
Forest stand type (Pine)	1.69	1.04	1.62	0.11	–0.36, 3.74	NA	NA	NA		NA	NA
Spatial scale (<5 ha)	–0.39	1.09	–0.36	0.72	–2.53, 1.74	NA	NA	NA		NA	NA
Spatial scale (5–30ha)	0.66	0.63	1.05	0.29	–0.58, 1.90	NA	NA	NA		NA	NA
Spatial scale (>50 ha)	–1.14	0.72	–1.58	0.11	–2.55, 0.27	NA	NA	NA		NA	NA
Tool used (Manual)	NA	NA	NA	NA	NA	–0.13	0.45	–0.30		0.77	–1.02, 0.75
Tool used (Natural)	NA	NA	NA	NA	NA	–0.42	0.67	–0.62		0.53	–1.73, 0.89
Invasive genus (Pooled)	NA	NA	NA	NA	NA	–0.86	1.14	–0.75		0.45	–3.09, 1.37
Invasive genus (*Rosa*)	NA	NA	NA	NA	NA	–2.03	1.37	–1.48		0.14	–4.72, 0.66
Test of Moderators											
*Q*_*M*_, df	30.89, 11					18.00, 8					
*p*‐value	0.001*					0.02*					
Test for Residual Heterogeneity											
*Q*_*E*_, df	308.28, 62					109.44, 27					
*p*‐value	<0.0001*					<0.0001*					

#### (ii) Invasive vegetation removal

Nine studies on the effect of invasive vegetation removal on tick density yielded 36 paired means. Five studies also included assessments of tick infection prevalence, but one did not provide any measure of precision to allow calculation of effect sizes, resulting in a sample size of 14 paired means. Invasive vegetation removal correlated with reduced tick density (estimated overall effect = 1.01, SE = 0.18, *p* < 0.0001), but not infection prevalence (estimated overall effect = 0.28, SE = 0.18, *p* = 0.11) (table 1, electronic supplementary materials, figures 4 and 5). This effect size was not only significant, but quite large. Hypothetically, removing invasive vegetation could reduce mean tick density from 25 ticks per 100 m^2^ (SD = 10) to 14.9 ticks per 100 m^2^ (an over 40% reduction) or, over an entire hectare, reduce this density by 1010 ticks.

As in the prescribed burn analysis, estimates of between-study heterogeneity were substantial for the invasive vegetation versus tick density model, but not for the tick infection prevalence model (table 1). Together, the moderators did significantly influence variation in effect sizes (*Q*_*M*_ = 18.00, *df* = 8, *p* = 0.02). The overall effect of invasive vegetation removal on tick density was moderated by tick life stage, with greater effects on adults than nymphs or all life stages combined (table 2). However, the effect size for nymphs was only moderate (effect = −0.35, SE = 0.12, *p* = 0.002), suggesting a limited ecologically meaningful difference between effects on adults versus nymphs. Additionally, the difference between adults versus pooled life stages was likely an artefact of data reporting (see §4). Tick genus, forest stand type, management tool used and invasive plant genus were not significant. This model explained 33% of between-study variance compared with the intercept-only model. Again, the test for residual heterogeneity was significant (*Q*_*E*_ = 109.44, *df* = 27, *p* < 0.0001), indicating other factors not considered in the model likely influenced the impact of invasive vegetation removal on tick density.

### (b) Literature review

#### (i) Prescribed burns and tick density and infection prevalence

Of the studies that examined the effects of prescribed burns on tick density (*n* = 9), seven documented lower tick density (*Dermacentor reticulatus, I. scapularis and A. americanum*) in burned areas and three documented either higher tick density (*A. americanum*) or no effect. One study documented both an immediate decrease and delayed increase in tick density (*A. americanum*) post-burn, depending on time since burn [15]. The authors speculated that several potential mechanisms could explain the link between prescribed burns and lower tick density. In particular, authors of three studies suggested that prescribed burns directly kill ticks [12,15,48]. Understory vegetation and leaf litter loss, and subsequent reduced microclimate humidity and tick moisture retention, may indirectly cause tick mortality [10,12,15,48], impair oviposition of engorged females or reduce abundance of oviposition sites or egg survival and viability [12]. While authors from multiple studies cited direct mortality (*n* = 4) and microclimate (*n* = 4) as mechanisms underlying the impact of fire on ticks, evidence for these causal relationships is nuanced. The effects of fire causing direct tick mortality may vary in magnitude depending on life stage; Davidson *et al*. [12] suggested that suppression of *A. americanum* larvae by fire is greater compared with that of adults and nymphs. Understory vegetation loss by fire also may reduce humidity in the microenvironment, indirectly causing tick desiccation [48]. Wildlife hosts for juvenile ticks may avoid burned areas, resulting in slow tick reintroductions and low tick densities [49], although larger hosts like white-tailed deer (*Odocoileus virginianus*) may return shortly after burns, potentially contributing to tick recolonization [14]. Gleim *et al*. [10] considered both host and microclimate mechanisms, finding stronger support for the latter as deer occurrence was not an important predictor of tick counts.

Of the studies that examined effects of prescribed burns on tick infection prevalence (*n* = 3), in burned areas, one documented lower endosymbiont (not pathogenic) *Rickettsia* spp. infection prevalence in *I. scapularis* [11], a second documented no difference in *Borrelia burgdorferi* infection prevalence in *I. scapularis* [48] and a third reported lower *Rickettsia* spp. infection prevalence in *A. americanum* but no difference in prevalence of *A. maculatum*, *D. variabilis* or *I. scapularis* infected with *Anaplasma* spp., *Borrelia* spp., *Ehrlichia* spp. or *Rickettsia* spp. [47]. Only two studies included discussions of mechanisms connecting prescribed burns and tick infection prevalence. Both discussed host mechanisms [47,48] and one also suggested a microclimate mechanism [47]. Tick-borne pathogen infection prevalence will be reduced if ticks derive more blood meals from less competent reservoir hosts like deer, and prescribed burns may influence whether these hosts are present owing to altered habitat [47]. Burns may also cause a disproportionate direct mortality of deer-derived ticks compared with mouse-derived ticks as the latter may be sequestered in mouse burrows and protected from the fire [48]. Gleim *et al*. [47] found no effect of prescribed burns on infection prevalence of bacteria pathogenic to humans but did find reduced non-pathogenic *Rickettsia* spp. infection prevalence. The authors suggest the post-burn microclimate’s higher temperature and lower humidity could affect the ability of ticks to maintain a *Rickettsia* infection because some *Rickettsia* species were unable to grow at high temperatures created in a laboratory setting [50].

#### (ii) Invasive vegetation removal and tick density and infection prevalence

Of the studies on the effects of invasive vegetation removal on tick density (*n* = 9), seven documented higher tick density in the presence of invasive vegetation [16–19,21,22,51], one documented lower tick density [51], and one [20] documented both reduced and increased tick density with invasive vegetation presence, depending on spatial scale. This last study found that, within the same forest fragment, *I. scapularis* nymph densities were higher in subplots containing *R. multiflora* than subplots where this invasive plant was absent, but in comparing entire invaded versus uninvaded forest fragments of between 6 and 16 ha in area, nymph densities were higher in forest fragments without *R. multiflora*. Most studies found that invasive vegetation removal reduced tick densities, possibly because invasive vegetation may improve host-seeking tick habitat by increasing microclimate humidity [17–19,22,52], thereby preventing desiccation and facilitating tick survival [53–57]. However, the effects of invasive vegetation on microclimate may depend upon invasive vegetation species. For example, while Williams & Ward [52] and Williams *et al*. [18] documented higher humidity in the presence of invasive *B. thunbergii*, Allan *et al*. [21] did not find evidence for this abiotic microclimate mechanism as tick survival remained constant between invasive *Lonicera maackii* presence and absence. Similarly, outside the context of tick-borne disease systems in temperate forests, some invasive woody shrubs like *Ligustrum sinense* maintained higher soil moisture in invaded versus uninvaded plots [58], while others, like the mat-forming perennial herb *Wedelia trilobata*, had no effect on soil moisture [59]. The presence of invasive vegetation may also increase host occurrence and consequently support higher tick densities through multiple pathways. Mice may seek out favourable foraging conditions within dense growths of invasive *B. thunbergii* owing to its abundant fruit serving as a potential food source [22]. Dense shrub thickets created by invasive *B. thunbergii* and *Lonicera* spp. may provide suitable cover and protection from predation for reproductive-stage hosts like deer [16,17,21] and house smaller hosts like birds and small mammals [17,22]. Moreover, abundant tick questing habitat within invasive vegetation may increase tick–host encounters and support higher tick densities [17,22,60].

Of the studies investigating effects of invasive vegetation removal on tick infection prevalence (*n* = 5), three documented lower prevalence of *B. burgdorferi* infection in *I. scapularis* with reduced invasive vegetation [22,23,52] and two documented either no effect on *Ehrlichia chaffeensis* or *E. ewingii* infection prevalence in *A. americanum* [21] or too low study-wide *B. burgdorferi* infection prevalence in *I. scapularis* for analysis [19]. Only three studies considered mechanisms driving infection prevalence specifically [21–23], all of which were host mechanisms. Invasive vegetation may provide suitable habitat for juvenile ticks and competent pathogen reservoirs, like small mammals and birds, aggregating and facilitating interactions between vectors and reservoir hosts [22,23]. Williams *et al*. [22] hypothesized that areas with invasive vegetation would have higher small mammal densities and tick infection prevalence, but instead only found higher mouse larval tick burdens in invasive-dominated understories and did not detect differences in mouse densities. Adalsteinsson *et al*. [23] reported a positive correlation between mouse abundance and tick infection prevalence, consistent with previous documentation of *I. scapularis* nymphal infection prevalence positively correlating with mouse density [61]. Allan *et al*. [21] found a greater density of infected nymphs with invasive plant presence, but this was driven largely by nymph density as they found no difference in nymphal infection prevalence. In this study, there was a correlation between the proportion of blood meals taken from deer hosts and plots invaded with bush honeysuckle, and the proportion of deer-derived blood meals in turn positively correlated with *E. ewingii* infection prevalence.

#### (iii) Timber harvesting and tick density and infection prevalence

Although only two studies examined the effects of timber harvesting on tick density [6,7], both of these documented reduced tick density post-harvest. Both speculated on potential microclimate and host mechanisms that could explain the link between timber harvesting and lower tick density. Forest stands harvested within the past 5 years had higher temperatures and vapour pressure deficits (VPD) in the microenvironment, which may decrease tick questing activity or longevity and inhibit ticks’ ability to acquire a blood meal, ultimately lowering the tick population size [6]. Both studies documented reduced mouse live capture rates in stands harvested within the past 2 [7] to 5 [6] years compared with stands with no recent harvest history, possibly owing to reduced foraging behaviour or predator avoidance in these exposed habitats, which may limit tick–host encounters and tick densities. Only one study reported statistical comparisons of questing tick infection prevalence, finding no effect of timber harvesting on infection prevalence. A lack of detectable differences in host diversity and community reservoir competence could explain the consistent tick infection prevalence across harvested and unharvested sites [6]. Additionally, a recent study conducted outside the time of this literature search found reduced tick densities in forests with a history of timber harvesting [8].

## 4. Discussion

To our knowledge, this is the first systematic review and meta-analysis to consider the net impacts of forest management practices on tick density and infection prevalence. As study characteristics such as sample size, tick genus and study location can contribute to substantial between-study heterogeneity among field studies, we standardized effect sizes to identify conditions under which active forest management can reduce key measures of tick-borne disease exposure risk in the environment. Most studies on the effects of prescribed burns or invasive vegetation removal on tick density found lower tick density in managed areas. Of the studies that did not find that prescribed burns correlated with lower tick density, a lack of effect may be owing to a low burn intensity [13] and/or because ticks were able to quickly reestablish burned areas after dropping from hosts, like white-tailed deer, that immigrated to post-burn areas to forage [13,15]. Years after the prescribed burn, new growth forage may improve in nutritional content and palatability, and decrease in physical defences, thereby attracting deer [13–15]. In some areas of Missouri [14] and Florida [62], deer returned almost immediately post-burn. Potentially, some burns may result in an initial reduction in tick density immediately post-burn, followed by a rebound in the tick population within 2 to 5 years to levels exceeding unburned control sites, if host activity is conducive to this recolonization [14,15]. Of the studies that did not find evidence of higher tick density in areas with invasive vegetation presence, some authors postulated that the presence of invasive plants may have *reduced* tick densities because of the plants’ chemical composition. For example, the polyphenols and cyanidin within the leaf litter of the invasive plant *Rhododendron ponticum* may reduce tick density [51]. In another study, the authors suspect they found lower tick densities in forest fragments invaded by invasive plants owing to unfavourable tick habitat caused by lower overall leaf litter volume, rather than owing to invasive plant presence itself [20]. Although too few studies have been conducted to date to carry out a meta-analysis on the effects of timber harvesting on tick density or infection prevalence with sufficient statistical power, the two studies that existed at the time of the literature search reported a consistent effect of timber harvesting reducing tick density [6,7].

Although we found that time since management only minimally influenced the effect of prescribed burning on tick density, there are studies that documented rebounds in *A. americanum* adult and nymph densities 2 to 5 years following prescribed burns [14,15]. Future studies should investigate possible effects beyond 5 to 10 years post-burn as prescribed burns may reduce tick density but require repeated application to continue to be effective over time. Beyond this single moderator, the combined influence of all moderators did significantly contribute to variation in effect sizes reported across studies. Cumulative effects from certain factors may contribute to how effectively prescribed burning alters tick density. For example, there may be varied effects on *Ixodes* spp. versus *Amblyomma* spp. because of differences between the two genera in behaviour and tolerance to desiccating conditions. Portions of *Amblyomma* populations may avoid direct mortality by fire during prescribed burns by descending into the forest floor detritus [14,63]. Additionally, compared with *I. scapularis*, *Amblyomma* species like *A. americanum* are more common in lower depths of leaf litter and more robust to higher temperatures, low humidity and moisture levels [64–67]. *Amblyomma*’s greater resilience to desiccating conditions [66] could potentially make this genus more tolerant of the unfavourable habitat created by prescribed burns than *Ixodes*, causing more substantial reductions in *Ixodes* density than in *Amblyomma* density post-burn. Given the ongoing spread of multiple *Amblyomma* species like *A. americanum* [68] and *A. maculatum* [69], we call for further investigation into whether certain management tools better mitigate *Amblyomma* versus *Ixodes* populations. Approaches may effectively reduce *Ixodes* densities but have a more limited impact on these invasive *Amblyomma* species. Even within a genus, the specific tick species might moderate management effects (e.g. prescribed burns reduced *Rickettsia* spp. infection prevalence in *A. americanum* but not in *A. maculatum* [47]). We were unable to parse this effect in our analyses owing to limited sample sizes for each species, but suggest future research compare the response of tick species within the same genus to these management practices. Future research could also investigate interacting effects between moderators, such as between time since management and tick genus. Owing to species-specific life cycles and apparent species-specific response to fire, understanding potential synergistic or antagonistic effects would deepen our mechanistic understanding of responses of tick populations to forest management.

We also found that tick life stage moderated the effect of invasive vegetation removal on tick density when all tick life stages were pooled versus when adults were considered separately, with invasive vegetation removal having a greater effect on adult densities than on the density of all combined life stages. However, this result is most likely an artefact of data reporting. Treatment effect is likely lessened when analysing pooled life stages because greater variation is introduced when multiple life stages are considered together owing to ecological differences between juveniles and adults. Future studies should analyse each life stage separately.

Multiple mechanisms may drive the relationship between these management practices and their relative efficacy in reducing tick density. Across all three types of active forest management, we found that a combination of microclimate and host mechanisms may explain the associations between management practices and tick density. The microclimate of host-seeking ticks’ habitat is well-established in the literature to affect tick survival and life history traits [56,70]. For example, *I. scapularis* nymphs’ optimal moulting temperature ranges between 16 and 30°C [71], and reduced relative humidity rates below 82% can inhibit *I. scapularis*’ survival [56]. Prescribed burns [10,12,15,48], the removal of invasive vegetation [17–19,22,52] and timber harvesting [6] may create less suitable tick microhabitat where microclimate temperature increases and humidity decreases.

Additionally, invasive vegetation may provide forage, fruit food sources, or suitable cover and protection from predation for hosts like deer and mice, increasing host occurrence in the presence of invasives and supporting higher tick densities [16,17,21,22]. Abundant questing habitat within invasive vegetation may increase tick–host encounters and support higher tick densities [17,22]. Removal of invasive vegetation, or areas naturally lacking invasive vegetation, can create less suitable tick and host habitat and cause lower tick density. Similarly, documented reductions in mouse capture rates in harvested stands [6,7] may result from reduced foraging behaviour owing to predator avoidance in these exposed habitats, subsequently leading to lower tick–host encounters and tick density.

Our inferences are limited by the information reported in the primary studies. For instance, multiple authors addressed the potential of varying burn intensities to have different effects on tick populations but there is yet to be a study that compares different intensities, and, furthermore, standardized measures of burn intensity are not yet established within tick-borne disease literature. One study defined a burn as low intensity because flame heights were < 1 m [13], while another considered a burn with flame heights of 0.6–0.9 m to be of moderate to severe intensity [9]. Gilliam *et al*. [13] found no effect of burning on the total *A. americanum* collected of all life stages, but speculated that the lack of effect may result from the low intensity of the burn. Many studies did not provide assessments of burn intensity and others used different metrics entirely to measure intensity, like large tree preservation but understory destruction [11] of shrubs less than 2 cm in diameter at breast height [48]. Gleim *et al*. [10] argued that tick population rebounds could occur after fire cessation when burns were not conducted like typical management burns. However, what constitutes a typical burn regime varies dramatically across the United States. For example, Allan [14] stated that burns studied in Missouri were applied every 3 to 5 years, which is the same time scale typically implemented in oak-hickory forests by management agencies, but in areas of Georgia and Florida, Gleim *et al*. [10] characterized their study sites to be typical of operational burns that occur over larger areas with repeated burns every 2 to 4 years. Therefore, we were unable to characterize moderating effects of varying burn intensities and encourage inclusion of standardized burn intensity metrics (e.g. mean flame height) in all future burn studies. It would be useful to know the burn intensity thresholds beyond which ticks cannot survive in order to compare the intensity needed to cause tick mortality versus intensities typically applied during management. More so, management agencies considering implementing prescribed burns to mitigate tick populations should consider such regional variability in burn regimes prior to application. Our study is also limited to examining the effects of forest management solely on questing ticks and does not consider on-host tick response. Potentially, on-host ticks could facilitate recovery of tick populations post-management. Future research could disentangle the consequences of prescribed burns, invasive vegetation removal and timber harvesting on questing ticks, as well as on-host ticks. Additionally, we included study accession number as a random effect in our models to account for non-independence of multiple paired means pulled from the same study. This may have limited our ability to detect influences from moderators that only varied between, and not within, studies. Including the random effect of study identifier accounted for between-study variation, which could diminish the detected effect of variables that vary only between studies, such as forest stand type. Forest stand type could contribute to the combined influence of moderators on the effect of prescribed burns on tick density, such as in oak-dominated versus mixed stands. For *I. scapularis*, there is high inter-annual variation in tick density in oak stands during mast versus non-mast years [72], and so prescribed burns may reduce tick densities more when applied during a non-mast year.

The sparse findings we found focusing on the effect of prescribed burns on tick infection prevalence (*n* = 6 paired means) showed no correlation, though we caution against generalizing these results with such a small sample size and potential for Type II error. Across studies of all three forest management practices, discussions of mechanisms to explain infection prevalence were limited as authors often addressed only predictors of tick density. Any predictors of infected ticks’ density still pertained to causes of tick density and did not specifically address drivers of infection prevalence alone. Discussion of ecological pathways to explain infection prevalence may be infrequent because few studies report significant differences in tick infection prevalence in managed versus unmanaged areas.

In summary, here we address a key forest management and public health policy question: can these common forest management practices be used as tools to mitigate hard-bodied tick densities and/or infection prevalence in temperate forests? Until now, this policy question remained unaddressed as there was no synthesis of prior field studies’ findings spanning a range of tick-borne disease systems that considered net effects of these three management practices. This meta-analysis confronts a significant knowledge gap in the study of anthropogenic land use change and tick ecology by deepening our understanding of the consequences for tick density and infection prevalence within disturbed temperate forests. These findings may help land managers, landowners and policy makers in shaping practices and policies. The social desirability of active forest management [5] like prescribed burns and invasive vegetation removal coupled with our findings demonstrate these tools as viable options for tick management, although further investigation is necessary to understand how varying levels of moderators impact the efficacy of management and overall effect. To further this field of study, we especially recommend that future research explores mechanisms for tick infection prevalence and, for prescribed burn studies, that some standardized measurement of burn intensity is employed, and more long-term studies are conducted to assess temporal fluctuations in tick populations post-burn.

## Data Availability

Data and code are included in electronic supplementary material File #7. Supplementary material is available online [73].
